# The forgotten story behind the first published retinal paintings

**DOI:** 10.1007/s00417-026-07196-2

**Published:** 2026-03-27

**Authors:** Jan E. E. Keunen, C. Richard Keeler, Camiel J. F. Boon, Phil W. Koken

**Affiliations:** 1 Department of Ophthalmology, Radboud University Medical Center, Nijmegen, the Netherlands; 2 Royal College of Ophthalmologists Museum and Library, London, UK; 3 Department of Ophthalmology, Amsterdam UMC, Amsterdam, the Netherlands; 4 Department of Radiation Oncology, Amsterdam UMC, Amsterdam, the Netherlands

**Keywords:** Ophthalmoscope, Retinal paintings, Adrien van Trigt

## Abstract

*****What is known***:**

• Helmholtz invented the ophthalmoscope in 1850 and published his observations in a monograph in 1851. However, he did not include an illustration of what he observed;

• The first published retinal paintings were created in 1853 by the artistically gifted physician Adrien Christophe van Trigt.

*****What is new***:**

• Shortly after Helmholtz’s publication of the ophthalmoscope, the Amsterdam instrument maker Epkens designed and constructed a table-model ophthalmoscope that left the dominant hand free;

• The ophthalmologist Donders in Utrecht recognized in Epkens’ table model ophthalmoscope the opportunity to produce retinal paintings for publication purposes. He recruited the artistically skilled physician Adrien van Trigt as PhD student to carry out this work;

• The collaboration of Donders, Van Trigt and Epkens resulted within 19 months following Helmholtz’ monograph, in the publication of retinal paintings – a remarkably fast achievement, given that retinal photography was not yet available.

## Abstract (structured)

**Purpose:**

To reconstruct the events that led to the first published retinal paintings following the invention of the ophthalmoscope, and to highlight their significance for the development of ophthalmic imaging.

**Methods:**

A historical analysis was performed based on primary sources, including Adrien van Trigt’s doctoral thesis (*De Speculo Oculi*, 1853), contemporaneous scientific publications, and archival material related to early ophthalmoscopy and retinal illustration.

**Results:**

Although Hermann von Helmholtz introduced the ophthalmoscope in 1850, no retinal images but descriptrions of what he observed accompanied his original publication. Within 19 months, the first retinal paintings were published by Adrien van Trigt. This achievement resulted from a collaboration between Franciscus Donders, who recognized the need for visual documentation, and the instrument maker Gerhard Epkens, who independently developed a table-mounted ophthalmoscope with improved illumination and stability. This design enabled simultaneous observation and drawing, allowing Van Trigt to produce the first coloured retinal illustrations, including representations of normal fundus anatomy and early depictions of retinal pathology such as retinitis pigmentosa.

**Conclusion:**

Achieved prior to the advent of retinal photography in1886, the first published retinal paintings represent a pivotal milestone in ophthalmology. They exemplify the interplay between clinical insight, technical innovation, and artistic expertise, and contributed significantly to the development of ophthalmic diagnostics and documentation.

## Introduction

The German physician and physicist Hermann von Helmholtz (1821–1894) invented the ophthalmoscope in 1850 and published his observations in a scientific monograph in 1851 [1, 2]. Since then, ophthalmologists worldwide use the ophthalmoscope on a daily basis.

Helmholtz was not the first to discover the principle of the ophthalmoscope. Jan Evangelista Purkinje (1787–1869) in 1823 and Charles Babbage (1791–1871) in 1847 already invented the principle of ophthalmoscopy [3]. Helmholtz immediately recognized and published the practical clinical value of the instrument. Although he described his observations in great detail, no retinal image was included in the monograph. This left ophthalmologists curious as to what could exactly be seen inside the eye. It took less than two years before the first retinal paintings were published in 1853, which was remarkably fast, given that medical photography did not yet exist.

Marking the 175th anniversary of Helmholtz’s monograph on ophthalmoscopy, we reconstruct the publication history of the first retinal paintings. This achievement was made in the Netherlands by teamwork of Franciscus Cornelis Donders (1818–1889), his PhD student Adrien Christophe Van Trigt (1825–1864) and the scientific instrument maker Gerhard Johann Abraham Epkens (1818–1873).

## Donders’ interest in Helmholtz’s ophthalmoscope

In 1847 physician Franciscus Donders was appointed as extraordinary professor at the University of Utrecht in the Netherlands. Among other medical disciplines he taught ophthalmology. In a small laboratory he started to examine refractive errors and accommodation in patients [4].

During his visit to the Great Exhibition in London in 1851, Donders learned from the German ophthalmologist Albrecht von Graefe (1828–1870) about the ophthalmoscope, prior to the publication of Helmholtz [5]. Donders had a keen interest in the new diagnostic instrument because it enabled him to study the inside of the eye of his patients with refraction anomalies non-invasively. Donders felt the urgency to share the news with his colleagues in the Netherlands: “*During my stay in London*,* I was informed by Von Graefe from Berlin that Helmholtz*,* professor of physiology at Königsberg*,* had found a means of observing the retina in another person’s eye. Because of the importance of this finding*,* I felt compelled to report it immediately to the Dutch medical public* [5].

Donders promptly ordered an ophthalmoscope from Helmholtz, but its delivery was delayed as Helmholtz was confronted with an unexpected surge in demand, receiving 18 orders within a short period. Fortunately for Donders, Von Graefe in Berlin, who was the first to use the ophthalmoscope routinely, send him a copy of the instrument early February 1852, which was manufactured by his instrument maker Dörffel (1810–1878) [6].

Meanwhile the scientific instrument maker Epkens in Amsterdam had also heard about the ophthalmoscope by Jan van Geuns (1808–1880), professor of pathology in Amsterdam [7]. It is likely that Van Geuns became aware of the ophthalmoscope following Donders’s public announcement [5]. Using the technical drawings in Helmholtz’s monograph, Epkens designed a new prototype ophthalmoscope. Instead of glass plates, which Helmholtz had used as reflector, Epkens chose a plano-mirror resulting in much better light output [8]. A small oval area of the metal layer was removed in the middle, creating a small glass window for examination by the observer. A brass tube with a positive converging lens was added between the light source (the flickering flame of an oil lamp) to focus the light source onto the plano-mirror. A movable disc allowed the observer to choose corrective lenses. Finally, Epkens placed the elongated instrument which cannot be held in the hand for reasons of stability on a stand, screwed to the edge of a Table [9].

**Fig. 1:**
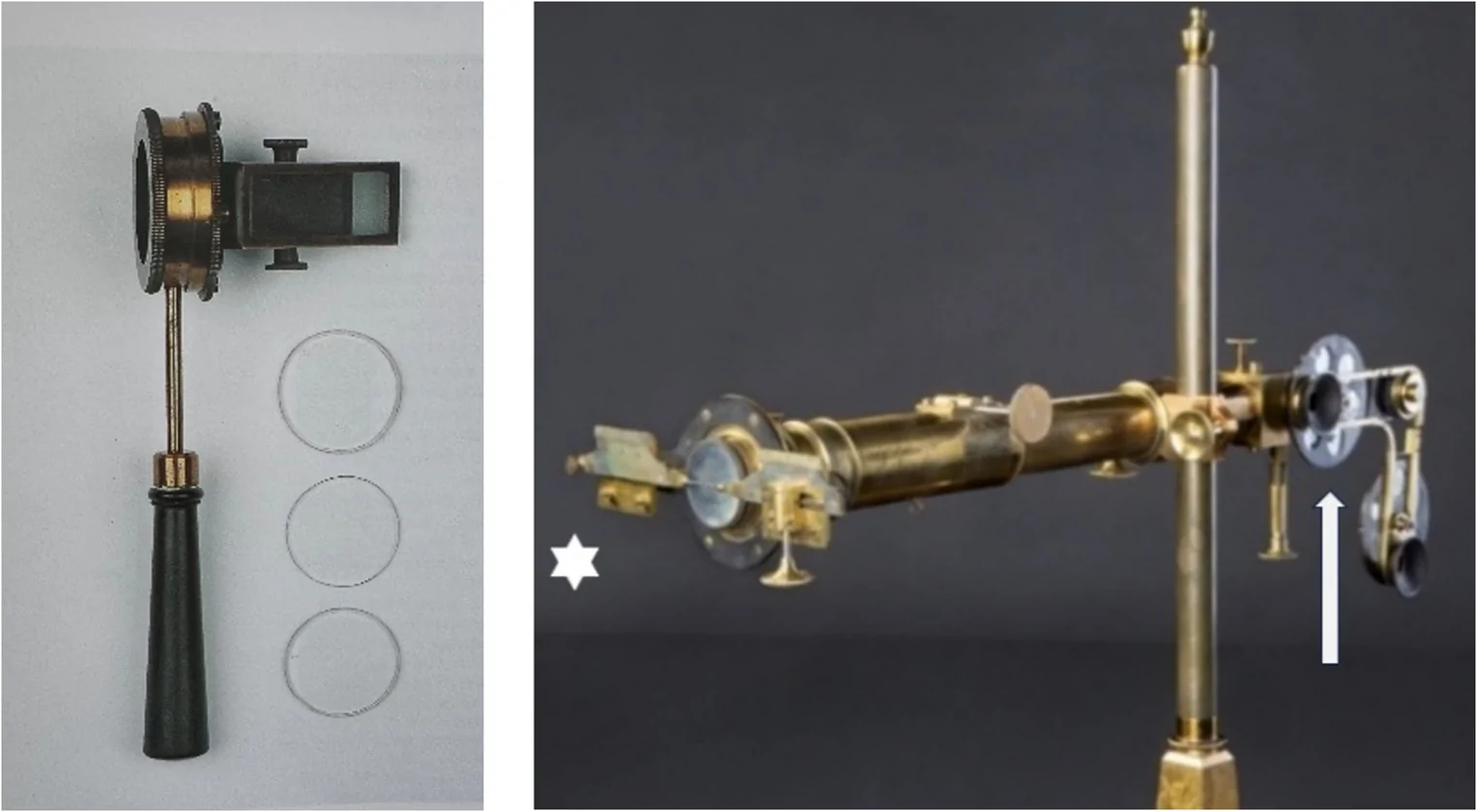
On the left an original handheld Helmholtz ophthalmoscope is shown (dated 1851–1852). Provenance: University Museum Utrecht, Utrecht, the Netherlands. On the right the Epkens-Donders table model ophthalmoscope designed by Epkens with improvements by Donders is shown, bearing the signature “G. Epkens & Co, Amsterdam” (dated 1852). On the left of the brass tube (white star) an oil lamp, which is not present here, was put on the table of which the flame served as light source. On the right (white arrow) a black eyepiece mounted on a square box from where the observer could see the fundus of the patient sitting at the opposite side. Provenance: Historic medical collection, Amsterdam UMC, Amsterdam, the Netherlands

Donders contacted Epkens in Amsterdam after hearing about this new prototype ophthalmoscope and suggested to add a micrometer in order to measure the diameter of retinal vessels. He also suggested to decrease the diameter of the mirror in order to reduce glare and to improve the mobility of the instrument enabling examination of a larger part of the retina [8, 9]. Donders preferred Epkens’ ophthalmoscope because of the considerably increased illumination of the retina and the short learning curve to use it compared to Helmholtz’s ophthalmoscope [9]. Finally, Donders added a rotating disk with positive lenses in order to observe structures at different levels into the eye [8, 9]. This made examination possible of the crystalline lens and vitreous body in a time that the slit-lamp did not yet exist. The original handheld Helmholtz’ ophthalmoscope and the Epkens-Donderstable model ophthalmoscope are shown in Fig. 1.

Den Tonkelaar et al. [10] stated that Donders commissioned Epkens to construct a table-mounted ophthalmoscope. This assertion was subsequently repeated by Muirhead [11]. On the basis of a careful review of the literature, we conclude that this interpretation is incorrect because Epkens independently designed a table-model ophthalmoscope [7–9]. On the left an original handheld Helmholtz’s ophthalmoscope is shown (dated 1851–1852).

## First published retinal paintings

Donders realized that retinal illustrations suitable for publication were lacking and decided to fill this gap. He cleverly took advantage of the fact that the table-model ophthalmoscope made it easier to produce fundus paintings as a standard practice, as it freed the dominant hand for drawing—an important benefit for publication purposes. He was therefore looking for a physician who could perform scientific ophthalmoscopic examinations while simultaneously producing accurate fundus paintings. Soon he managed to employ the physician Adrien van Trigt as PhD student, whose talents for drawing were particularly helpful [12, 13]. Van Trigt painted what he observed and noted a short patient history, usually in a few sentences. In his own words: *With this tool*,* one can easily observe the retina*,* sitting quietly opposite each other* [9]. In a lecture for the Royal Academy of Sciences on March 26 in 1853, Donders stated that: “*Mr. van Trigt took a very active part in the investigations … and made the drawings with care”* [8]. Information on how Van Trigt precisely made the retinal paintings is not known because none of the original paintings have been preserved. What we do know is that atropine sulfate eye drops were used for pupil dilatation in a 1,8% concentration [9].

On 16 June 1853, Van Trigt defended his PhD thesis, entitled “*Dissertatio ophthalmologica inauguralis de speculo oculi*” [9]. Its structure is divided in a short introduction, three chapters and a concise summary with the opening sentence: *We look back with satisfaction at what the new tool for science and practice delivers*. It is noteworthy that Van Trigt did not state anywhere that he had made the retinal paintings himself.

**Fig. 2:**
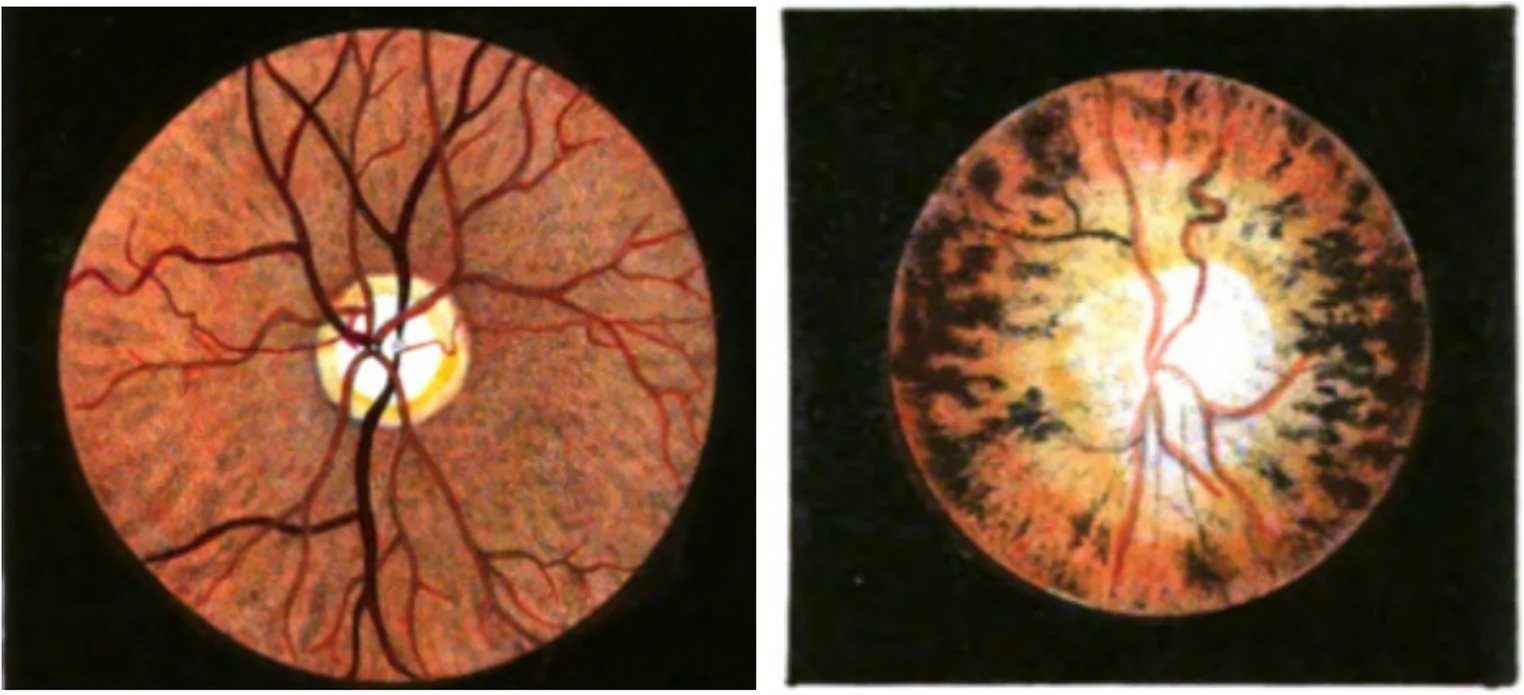
The retinal paintings of Van Trigt were published as coloured lithographs. A: The fundus of a healthy person. B: The fundus of male patient number XVII, aged 42, with progressive visual loss since 20 years and tunnel vision. The fundus is described as “a retina with strong pigment development”. This is the first published retinal illustration image of a retinitis pigmentosa patient. Only four years later Donders coined the term retinitis pigmentosa in 1857 [14]. Provenance: Medical and dental collections, Utrecht University, The Netherlands

The first chapter describes the history of ophthalmoscopy and the ophthalmoscopes of Helmholtz and Epkens. The second chapter is on observations in healthy animal and human eyes and the third chapter is about sick animal and human eyes. Dogs, cats, a sheep, rabbits and an owl were used as animals. Of 27 patients the clinical picture and ophthalmoscopic findings are briefly described. Two coloured lithographs based on Van Trigt’s retinal paintings show coloured images of the fundus in a healthy subject and six images of the 27 described patients, including the first coloured image of a healthy fundus, the first retinal detachment illustration and the fundus of a patient with the at that time unknown disease retinitis pigmentosa. (Fig. 2) [9]. Only four years later Donders went deeper in classifying what was observed in this patient and coined the term “retinitis pigmentosa” in 1857 [14]. Muirhead described what Van Trigt and Donders in 1853 were able to see and learned [11].

Van Trigt obtained the *magna cum laude* honour for his thesis [4]. A Dutch translation was published simultaneously in two scientific journals [15, 16]. An improved German edition appeared in 1854 through which the thesis gained wider recognition [12, 17].

The news that Donders could examine the interior of a patient’s eye resulted in a large influx of eye patients referred from all over the country to Utrecht. This led to the establishment of the first eye hospital in the Netherlands in 1858 after a crowdfunding campaign by Donders.

## Brief history of early retinal atlases

Two years after Van Trigt’s thesis was published, more retinal illustrations were published by Jaeger (1818–1884) in Vienna [12, 18].

In 1863 the first retina atlas was published in Von Graefe’s clinic in Berlin by Richard Liebreich (1830–1917), an ophthalmologist with artistic skills. He had learned ophthalmoscopy under Helmholtz’s supervision [12, 19]. His “Atlas der Ophthalmoscopie” included 57 high quality coloured lithographs of the retina, 12 of which were paintings by Liebreich. Editions appeared in English, French and Spanish [20]. Shortly thereafter followed an atlas containing 29 colour lithograph plates made from retinal paintings by Jaeger [12, 21]. A second edition contained already 73 coloured plates.

Over the next 60 years more than 50 ophthalmic atlases were published, containing an increasing number of plates depicting retinal disorders. The demand for retinal paintings to be included in ophthalmic textbooks increased as did the ophthalmologist’s understanding of the many retinal conditions [13]. A subsequent development was the publication of the first retinal photograph of a human eye in 1886 by Jackman and Webster [13]. Despite its limited image quality, this photograph marked a significant advance in the documentation of retinal images.

## Ophthalmic illustrator Adrien van Trigt

Adrien van Trigt’s life is briefly described by Muirhead [11]. Newly found biographical data revealed, among others, that Van Trigt was marked myopic. This made it easier for him to use the ophthalmoscope [9]. Van Trigt inherited his artistic skills from his father, who was a surgeon in Dordrecht (the Netherlands). They both had a strong interest in ornithology and made high quality, detailed watercolours of birds.

**Fig. 3:**
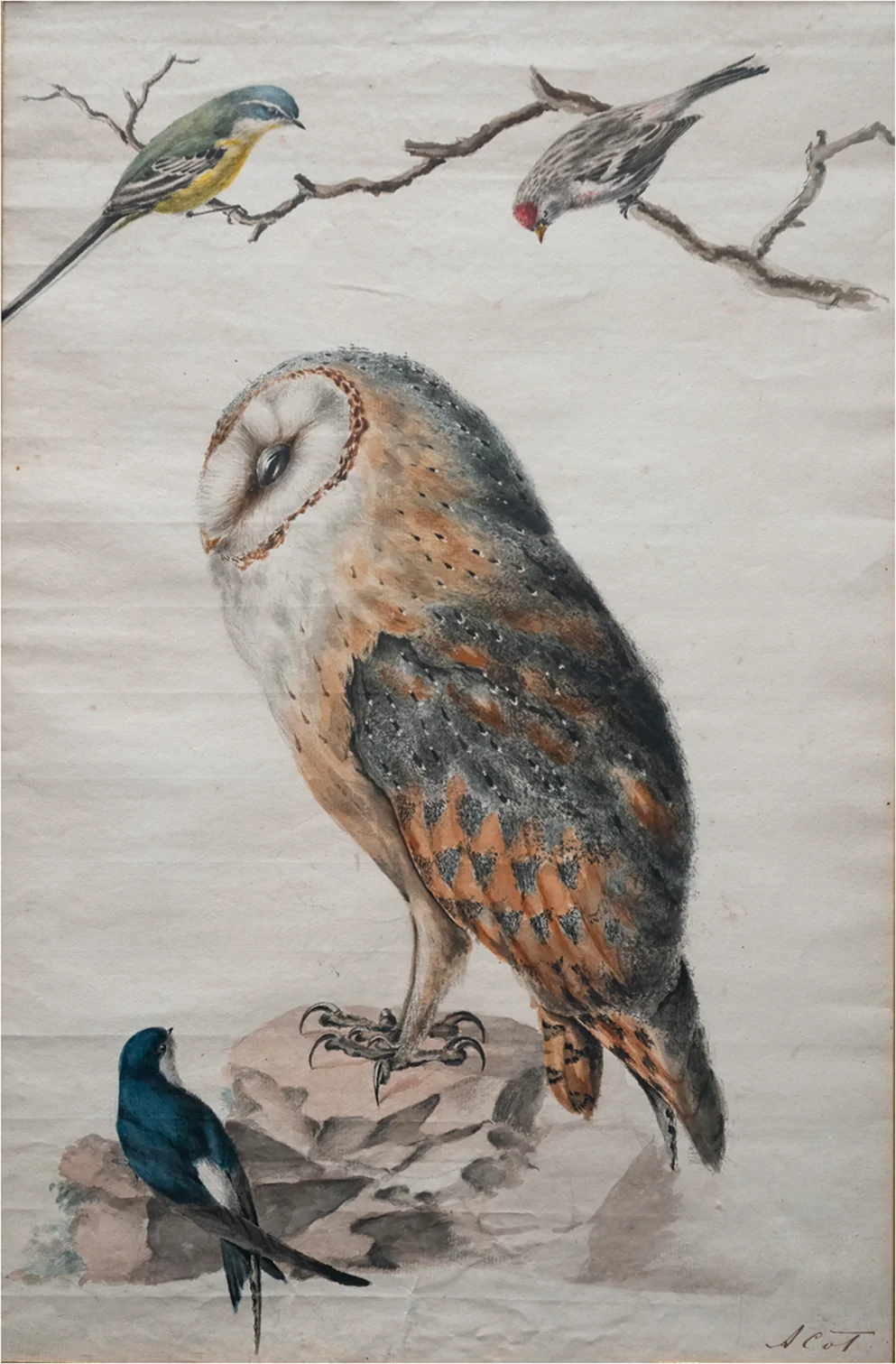
Adrian van Trigt’s watercolour of a Barn Owl, surrounded by (top left to bottom) a Yellow Wagtail, a Common Redpoll and a House Martin. Signed with the initials “AC v T”. A description of the fundus of an owl is included in Van Trigt’s thesis. Provenance: Private collection of the corresponding author

After completing his PhD trajectory in ophthalmology, Van Trigt embarked on a new medical career in venereology and dermatology in Amsterdam. Such a change in professional focus was not unusual among Donders’s 44 PhD candidates in ophthalmology [4]. Schauenburg and Donders explicitly acknowledged Van Trigt’s merits in the expanded German edition of his thesis [17]. A conflict between Donders and Van Trigt causing the change in Van Trigt’s career seems unlikely. In Amsterdam Van Trigt lived secluded amidst a small circle of close friends. His colleague Jan George Maria Hanlo (1831–1897) described his friend Adrien van Trigt as a very modest man with many talents, who suffered from an inferiority complex [22]. The promising life and career of Adrien van Trigt ended abruptly in 1864 at the age of 39 as a result of sudden death due to an apoplexia [23]. In an obituary and in a commemorative book containing Van Trigt’s unpublished lectures, which appeared after his untimely death, surprisingly his important contribution as creator of the first published retinal paintings was not mentioned [22, 24]. Even his close friend Willem Marius Gunning (1834–1912), who became professor of ophthalmology in Amsterdam in 1877, appears not to have recognized the significance of Van Trigt’s retinal paintings as a major contribution to international ophthalmology after his death. It was not until the golden jubilee of the ophthalmoscope in 1901 that professor Herman Snellen (1834–1908), who had worked with Van Trigt under Donders’ supervision in Utrecht during the 1850s, publicly reaffirmed Van Trigt’s achievements as an ophthalmic illustrator [25]. In 1967, Duke-Elder wrote in his *System of Ophthalmology* that Van Trigt published the world’s first printed coloured illustration of the fundus of the eye [20]. The artistic legacy of Adrien van Trigt lives on in a limited number of water colours of birds, mainly in private collections. Figure 3 illustrates an example of his artistic craftsmanship outside ophthalmology, reflecting his interest in ornithology.

## Summary

The invention of the ophthalmoscope in 1850 made it possible to visualise the interior of the living eye. This innovation rapidly expanded knowledge of ocular anatomy and physiology and soon underscored the importance of documenting the retina in both normal and pathological states [26].

Early retinal images were produced as retinal paintings, created under challenging conditions using primitive ophthalmoscopes and the unstable illumination of oil lamps. These retinal paintings were subsequently reproduced as coloured lithographs (chromolithographs) and printed as plates in retinal atlases [13]. Such atlases played a central role in the development of ophthalmology and contributed significantly to its emergence as a distinct medical specialty [26]. Retinal paintings remained the principal means of retinal documentation until 1886, when the first successful fundus photographs were produced [13, 26].

The teamwork between Donders, Epkens, and Van Trigt, which led to the first published retinal paintings, exemplifies the interplay between clinical observation, artistic skill, technical innovation, and scientific ambition that characterised the early retinal documentation of the fundus of the eye [26]. In contrast to modern high-resolution digital fundus imaging, available at the push of a button, their collaboration resulted in the publication of retinal images within only 19 months after Helmholtz’s monograph on ophthalmoscopy.

## Data Availability

No datasets were generated or analysed during the current study.
